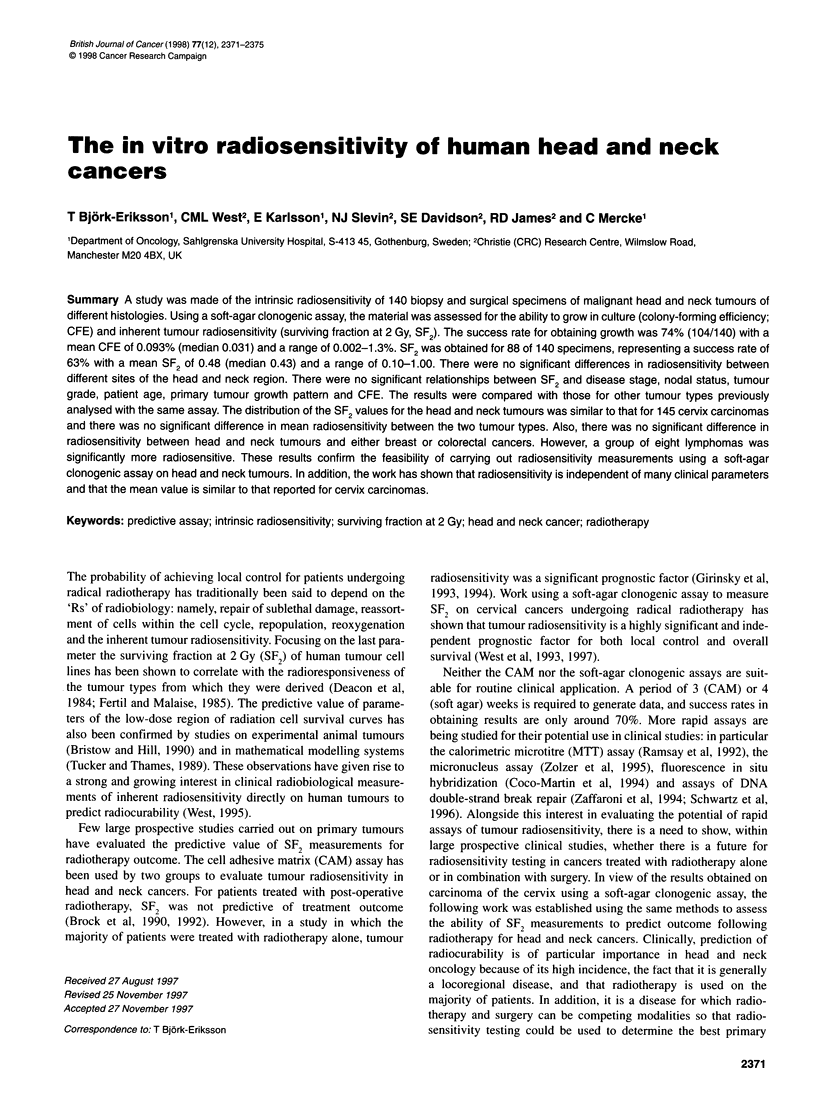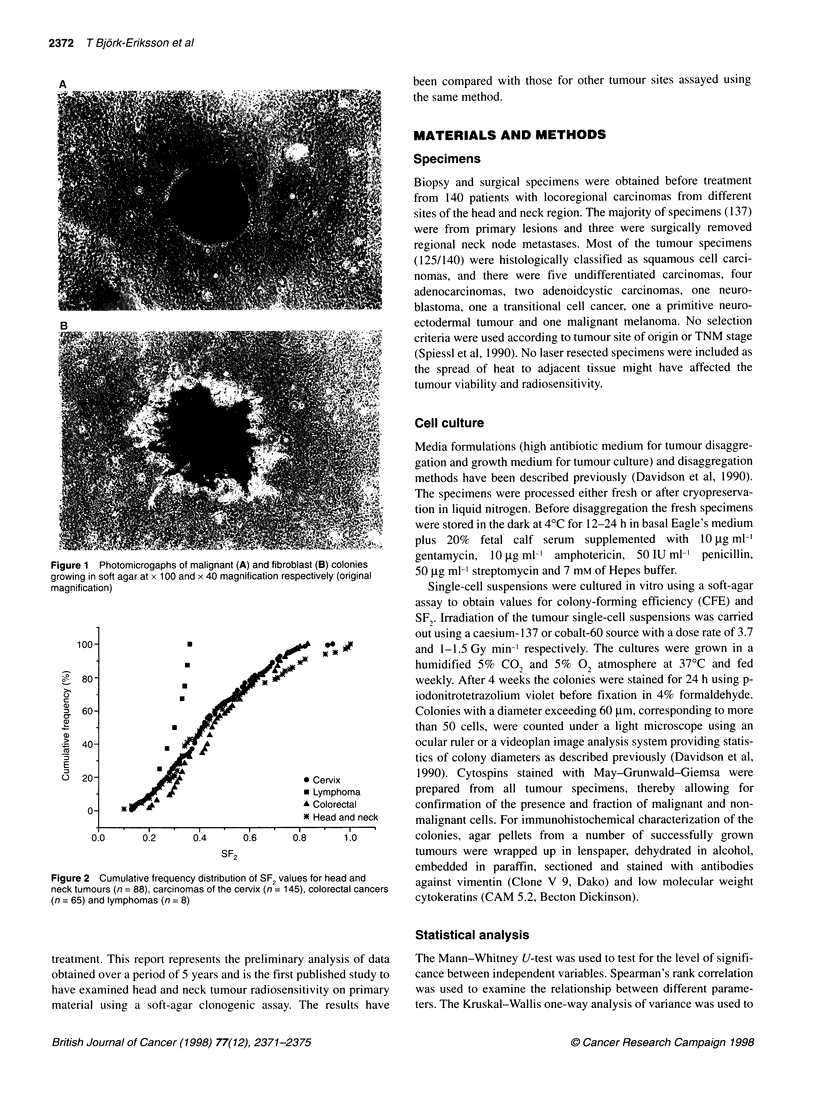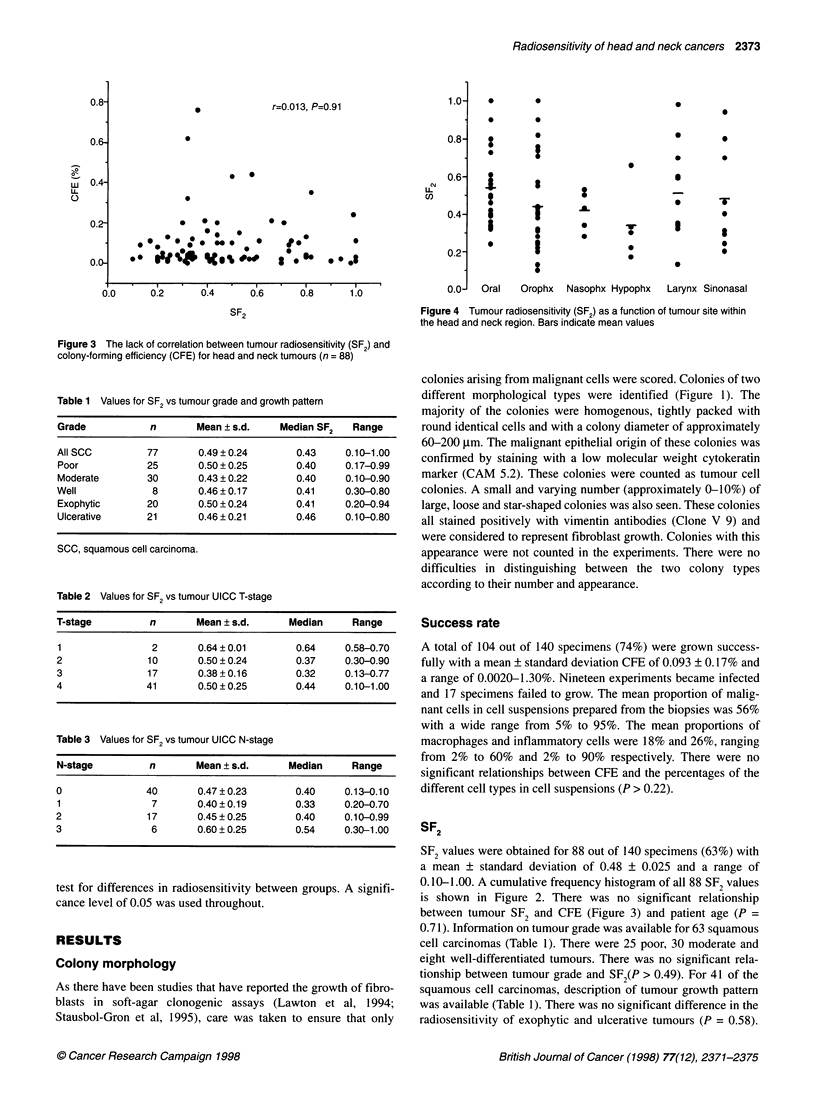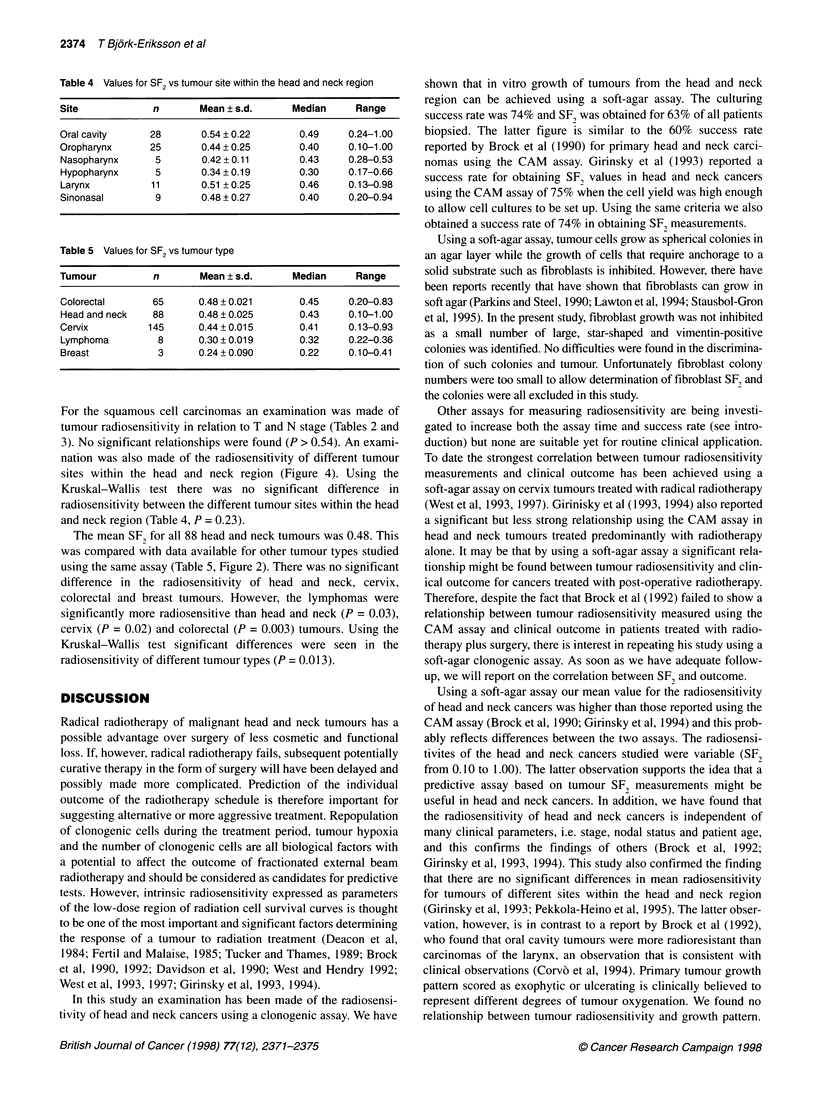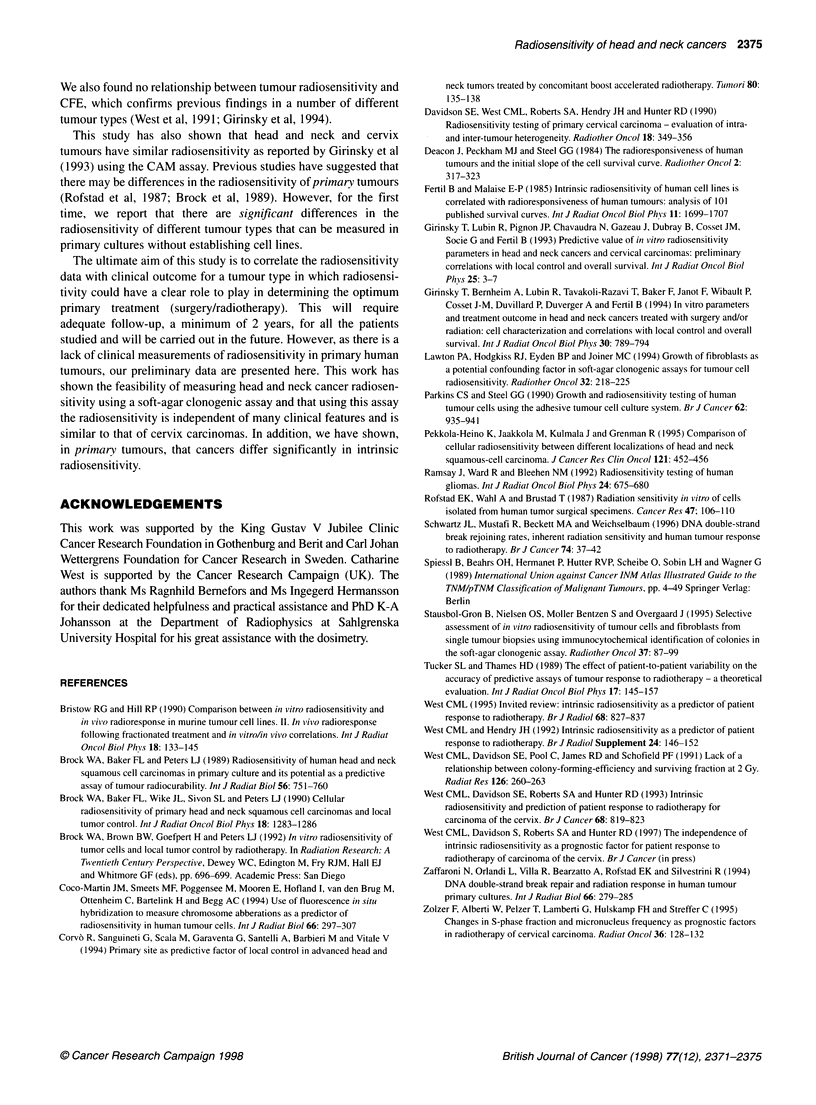# The in vitro radiosensitivity of human head and neck cancers.

**DOI:** 10.1038/bjc.1998.394

**Published:** 1998-06

**Authors:** T. BjÃ¶rk-Eriksson, C. M. West, E. Karlsson, N. J. Slevin, S. E. Davidson, R. D. James, C. Mercke

**Affiliations:** Department of Oncology, Sahlgrenska University Hospital, Gothenburg, Sweden.

## Abstract

**Images:**


British Journal of Cancer (1998) 77(12), 2371–2375
© 1998 Cancer Research Campaign

### The in vitro radiosensitivity of human head and neck cancers

**T Björk-Eriksson[1], CML West[2], E Karlsson[1], NJ Slevin[2], SE Davidson[2], RD James[2] and C Mercke[1]**

[1]Department of Oncology, Sahlgrenska University Hospital, S-413 45, Gothenburg, Sweden; [2]Christie (CRC) Research Centre, Wilmslow Road, Manchester M20 4BX, UK

**Summary** A study was made of the intrinsic radiosensitivity of 140 biopsy and surgical specimens of malignant head and neck tumours of different histologies. Using a soft-agar clonogenic assay, the material was assessed for the ability to grow in culture (colony-forming efficiency; CFE) and inherent tumour radiosensitivity (surviving fraction at 2 Gy, $SF_2$). The success rate for obtaining growth was 74% (104/140) with a mean CFE of 0.093% (median 0.031) and a range of 0.002–1.3%. $SF_2$ was obtained for 88 of 140 specimens, representing a success rate of 63% with a mean $SF_2$ of 0.48 (median 0.43) and a range of 0.10–1.00. There were no significant differences in radiosensitivity between different sites of the head and neck region. There were no significant relationships between $SF_2$ and disease stage, nodal status, tumour grade, patient age, primary tumour growth pattern and CFE. The results were compared with those for other tumour types previously analysed with the same assay. The distribution of the $SF_2$ values for the head and neck tumours was similar to that for 145 cervix carcinomas and there was no significant difference in mean radiosensitivity between the two tumour types. Also, there was no significant difference in radiosensitivity between head and neck tumours and either breast or colorectal cancers. However, a group of eight lymphomas was significantly more radiosensitive. These results confirm the feasibility of carrying out radiosensitivity measurements using a soft-agar clonogenic assay on head and neck tumours. In addition, the work has shown that radiosensitivity is independent of many clinical parameters and that the mean value is similar to that reported for cervix carcinomas.

**Keywords:** predictive assay; intrinsic radiosensitivity; surviving fraction at 2 Gy; head and neck cancer; radiotherapy

The probability of achieving local control for patients undergoing radical radiotherapy has traditionally been said to depend on the 'Rs' of radiobiology: namely, repair of sublethal damage, reassortment of cells within the cell cycle, repopulation, reoxygenation and the inherent tumour radiosensitivity. Focusing on the last parameter the surviving fraction at 2 Gy ($SF_2$) of human tumour cell lines has been shown to correlate with the radioresponsiveness of the tumour types from which they were derived (Deacon et al, 1984; Fertil and Malaise, 1985). The predictive value of parameters of the low-dose region of radiation cell survival curves has also been confirmed by studies on experimental animal tumours (Bristow and Hill, 1990) and in mathematical modelling systems (Tucker and Thames, 1989). These observations have given rise to a strong and growing interest in clinical radiobiological measurements of inherent radiosensitivity directly on human tumours to predict radiocurability (West, 1995).

Few large prospective studies carried out on primary tumours have evaluated the predictive value of $SF_2$ measurements for radiotherapy outcome. The cell adhesive matrix (CAM) assay has been used by two groups to evaluate tumour radiosensitivity in head and neck cancers. For patients treated with post-operative radiotherapy, $SF_2$ was not predictive of treatment outcome (Brock et al, 1990, 1992). However, in a study in which the majority of patients were treated with radiotherapy alone, tumour radiosensitivity was a significant prognostic factor (Girinsky et al, 1993, 1994). Work using a soft-agar clonogenic assay to measure $SF_2$ on cervical cancers undergoing radical radiotherapy has shown that tumour radiosensitivity is a highly significant and independent prognostic factor for both local control and overall survival (West et al, 1993, 1997).

Neither the CAM nor the soft-agar clonogenic assays are suitable for routine clinical application. A period of 3 (CAM) or 4 (soft agar) weeks is required to generate data, and success rates in obtaining results are only around 70%. More rapid assays are being studied for their potential use in clinical studies: in particular the calorimetric microtitre (MTT) assay (Ramsay et al, 1992), the micronucleus assay (Zolzer et al, 1995), fluorescence in situ hybridization (Coco-Martin et al, 1994) and assays of DNA double-strand break repair (Zaffaroni et al, 1994; Schwartz et al, 1996). Alongside this interest in evaluating the potential of rapid assays of tumour radiosensitivity, there is a need to show, within large prospective clinical studies, whether there is a future for radiosensitivity testing in cancers treated with radiotherapy alone or in combination with surgery. In view of the results obtained on carcinoma of the cervix using a soft-agar clonogenic assay, the following work was established using the same methods to assess the ability of $SF_2$ measurements to predict outcome following radiotherapy for head and neck cancers. Clinically, prediction of radiocurability is of particular importance in head and neck oncology because of its high incidence, the fact that it is generally a locoregional disease, and that radiotherapy is used on the majority of patients. In addition, it is a disease for which radiotherapy and surgery can be competing modalities so that radiosensitivity testing could be used to determine the best primary

*Received 27 August 1997*
*Revised 25 November 1997*
*Accepted 27 November 1997*

*Correspondence to:* T Björk-Eriksson

**Figure 1** Photomicrogaphs of malignant (**A**) and fibroblast (**B**) colonies growing in soft agar at × 100 and × 40 magnification respectively (original magnification)

**Figure 2** Cumulative frequency distribution of $SF_2$ values for head and neck tumours ($n$ = 88), carcinomas of the cervix ($n$ = 145), colorectal cancers ($n$ = 65) and lymphomas ($n$ = 8)

treatment. This report represents the preliminary analysis of data obtained over a period of 5 years and is the first published study to have examined head and neck tumour radiosensitivity on primary material using a soft-agar clonogenic assay. The results have been compared with those for other tumour sites assayed using the same method.

### MATERIALS AND METHODS

### Specimens

Biopsy and surgical specimens were obtained before treatment from 140 patients with locoregional carcinomas from different sites of the head and neck region. The majority of specimens (137) were from primary lesions and three were surgically removed regional neck node metastases. Most of the tumour specimens (125/140) were histologically classified as squamous cell carcinomas, and there were five undifferentiated carcinomas, four adenocarcinomas, two adenoidcystic carcinomas, one neuroblastoma, one a transitional cell cancer, one a primitive neuroectodermal tumour and one malignant melanoma. No selection criteria were used according to tumour site of origin or TNM stage (Spiessl et al, 1990). No laser resected specimens were included as the spread of heat to adjacent tissue might have affected the tumour viability and radiosensitivity.

### Cell culture

Media formulations (high antibiotic medium for tumour disaggregation and growth medium for tumour culture) and disaggregation methods have been described previously (Davidson et al, 1990). The specimens were processed either fresh or after cryopreservation in liquid nitrogen. Before disaggregation the fresh specimens were stored in the dark at 4°C for 12–24 h in basal Eagle's medium plus 20% fetal calf serum supplemented with 10 μg $ml^{-1}$ gentamycin, 10 μg $ml^{-1}$ amphotericin, 50 IU $ml^{-1}$ penicillin, 50 μg $ml^{-1}$ streptomycin and 7 mM of Hepes buffer.

Single-cell suspensions were cultured in vitro using a soft-agar assay to obtain values for colony-forming efficiency (CFE) and $SF_2$. Irradiation of the tumour single-cell suspensions was carried out using a caesium-137 or cobalt-60 source with a dose rate of 3.7 and 1–1.5 Gy $min^{-1}$ respectively. The cultures were grown in a humidified 5% $CO_2$ and 5% $O_2$ atmosphere at 37°C and fed weekly. After 4 weeks the colonies were stained for 24 h using p-iodonitrotetrazolium violet before fixation in 4% formaldehyde. Colonies with a diameter exceeding 60 μm, corresponding to more than 50 cells, were counted under a light microscope using an ocular ruler or a videoplan image analysis system providing statistics of colony diameters as described previously (Davidson et al, 1990). Cytospins stained with May–Grunwald–Giemsa were prepared from all tumour specimens, thereby allowing for confirmation of the presence and fraction of malignant and non-malignant cells. For immunohistochemical characterization of the colonies, agar pellets from a number of successfully grown tumours were wrapped up in lenspaper, dehydrated in alcohol, embedded in paraffin, sectioned and stained with antibodies against vimentin (Clone V 9, Dako) and low molecular weight cytokeratins (CAM 5.2, Becton Dickinson).

### Statistical analysis

The Mann–Whitney *U*-test was used to test for the level of significance between independent variables. Spearman's rank correlation was used to examine the relationship between different parameters. The Kruskal–Wallis one-way analysis of variance was used to

© Cancer Research Campaign 1998

**Figure 3** The lack of correlation between tumour radiosensitivity ($SF_2$) and colony-forming efficiency (CFE) for head and neck tumours ($n$ = 88)

**Figure 4** Tumour radiosensitivity ($SF_2$) as a function of tumour site within the head and neck region. Bars indicate mean values

**Table 1** Values for $SF_2$ vs tumour grade and growth pattern

| Grade | $n$ | Mean ± s.d. | Median $SF_2$ | Range |
|---|---|---|---|---|
| All SCC | 77 | 0.49 ± 0.24 | 0.43 | 0.10–1.00 |
| Poor | 25 | 0.50 ± 0.25 | 0.40 | 0.17–0.99 |
| Moderate | 30 | 0.43 ± 0.22 | 0.40 | 0.10–0.90 |
| Well | 8 | 0.46 ± 0.17 | 0.41 | 0.30–0.80 |
| Exophytic | 20 | 0.50 ± 0.24 | 0.41 | 0.20–0.94 |
| Ulcerative | 21 | 0.46 ± 0.21 | 0.46 | 0.10–0.80 |

SCC, squamous cell carcinoma.

**Table 2** Values for $SF_2$ vs tumour UICC T-stage

| T-stage | $n$ | Mean ± s.d. | Median | Range |
|---|---|---|---|---|
| 1 | 2 | 0.64 ± 0.01 | 0.64 | 0.58–0.70 |
| 2 | 10 | 0.50 ± 0.24 | 0.37 | 0.30–0.90 |
| 3 | 17 | 0.38 ± 0.16 | 0.32 | 0.13–0.77 |
| 4 | 41 | 0.50 ± 0.25 | 0.44 | 0.10–1.00 |

**Table 3** Values for $SF_2$ vs tumour UICC N-stage

| N-stage | $n$ | Mean ± s.d. | Median | Range |
|---|---|---|---|---|
| 0 | 40 | 0.47 ± 0.23 | 0.40 | 0.13–0.10 |
| 1 | 7 | 0.40 ± 0.19 | 0.33 | 0.20–0.70 |
| 2 | 17 | 0.45 ± 0.25 | 0.40 | 0.10–0.99 |
| 3 | 6 | 0.60 ± 0.25 | 0.54 | 0.30–1.00 |

test for differences in radiosensitivity between groups. A significance level of 0.05 was used throughout.

### RESULTS

### Colony morphology

As there have been studies that have reported the growth of fibroblasts in soft-agar clonogenic assays (Lawton et al, 1994; Stausbol-Gron et al, 1995), care was taken to ensure that only colonies arising from malignant cells were scored. Colonies of two different morphological types were identified (Figure 1). The majority of the colonies were homogenous, tightly packed with round identical cells and with a colony diameter of approximately 60–200 μm. The malignant epithelial origin of these colonies was confirmed by staining with a low molecular weight cytokeratin marker (CAM 5.2). These colonies were counted as tumour cell colonies. A small and varying number (approximately 0–10%) of large, loose and star-shaped colonies was also seen. These colonies all stained positively with vimentin antibodies (Clone V 9) and were considered to represent fibroblast growth. Colonies with this appearance were not counted in the experiments. There were no difficulties in distinguishing between the two colony types according to their number and appearance.

### Success rate

A total of 104 out of 140 specimens (74%) were grown successfully with a mean ± standard deviation CFE of 0.093 ± 0.17% and a range of 0.0020–1.30%. Nineteen experiments became infected and 17 specimens failed to grow. The mean proportion of malignant cells in cell suspensions prepared from the biopsies was 56% with a wide range from 5% to 95%. The mean proportions of macrophages and inflammatory cells were 18% and 26%, ranging from 2% to 60% and 2% to 90% respectively. There were no significant relationships between CFE and the percentages of the different cell types in cell suspensions ($P > 0.22$).

### $SF_2$

$SF_2$ values were obtained for 88 out of 140 specimens (63%) with a mean ± standard deviation of 0.48 ± 0.025 and a range of 0.10–1.00. A cumulative frequency histogram of all 88 $SF_2$ values is shown in Figure 2. There was no significant relationship between tumour $SF_2$ and CFE (Figure 3) and patient age ($P$ = 0.71). Information on tumour grade was available for 63 squamous cell carcinomas (Table 1). There were 25 poor, 30 moderate and eight well-differentiated tumours. There was no significant relationship between tumour grade and $SF_2$($P > 0.49$). For 41 of the squamous cell carcinomas, description of tumour growth pattern was available (Table 1). There was no significant difference in the radiosensitivity of exophytic and ulcerative tumours ($P$ = 0.58).

© Cancer Research Campaign 1998

**Table 4** Values for $SF_2$ vs tumour site within the head and neck region

| Site | n | Mean ± s.d. | Median | Range |
|---|---|---|---|---|
| Oral cavity | 28 | 0.54 ± 0.22 | 0.49 | 0.24–1.00 |
| Oropharynx | 25 | 0.44 ± 0.25 | 0.40 | 0.10–1.00 |
| Nasopharynx | 5 | 0.42 ± 0.11 | 0.43 | 0.28–0.53 |
| Hypopharynx | 5 | 0.34 ± 0.19 | 0.30 | 0.17–0.66 |
| Larynx | 11 | 0.51 ± 0.25 | 0.46 | 0.13–0.98 |
| Sinonasal | 9 | 0.48 ± 0.27 | 0.40 | 0.20–0.94 |

**Table 5** Values for $SF_2$ vs tumour type

| Tumour | n | Mean ± s.d. | Median | Range |
|---|---|---|---|---|
| Colorectal | 65 | 0.48 ± 0.021 | 0.45 | 0.20–0.83 |
| Head and neck | 88 | 0.48 ± 0.025 | 0.43 | 0.10–1.00 |
| Cervix | 145 | 0.44 ± 0.015 | 0.41 | 0.13–0.93 |
| Lymphoma | 8 | 0.30 ± 0.019 | 0.32 | 0.22–0.36 |
| Breast | 3 | 0.24 ± 0.090 | 0.22 | 0.10–0.41 |

For the squamous cell carcinomas an examination was made of tumour radiosensitivity in relation to T and N stage (Tables 2 and 3). No significant relationships were found ($P > 0.54$). An examination was also made of the radiosensitivity of different tumour sites within the head and neck region (Figure 4). Using the Kruskal–Wallis test there was no significant difference in radiosensitivity between the different tumour sites within the head and neck region (Table 4, $P = 0.23$).

The mean $SF_2$ for all 88 head and neck tumours was 0.48. This was compared with data available for other tumour types studied using the same assay (Table 5, Figure 2). There was no significant difference in the radiosensitivity of head and neck, cervix, colorectal and breast tumours. However, the lymphomas were significantly more radiosensitive than head and neck ($P = 0.03$), cervix ($P = 0.02$) and colorectal ($P = 0.003$) tumours. Using the Kruskal–Wallis test significant differences were seen in the radiosensitivity of different tumour types ($P = 0.013$).

### DISCUSSION

Radical radiotherapy of malignant head and neck tumours has a possible advantage over surgery of less cosmetic and functional loss. If, however, radical radiotherapy fails, subsequent potentially curative therapy in the form of surgery will have been delayed and possibly made more complicated. Prediction of the individual outcome of the radiotherapy schedule is therefore important for suggesting alternative or more aggressive treatment. Repopulation of clonogenic cells during the treatment period, tumour hypoxia and the number of clonogenic cells are all biological factors with a potential to affect the outcome of fractionated external beam radiotherapy and should be considered as candidates for predictive tests. However, intrinsic radiosensitivity expressed as parameters of the low-dose region of radiation cell survival curves is thought to be one of the most important and significant factors determining the response of a tumour to radiation treatment (Deacon et al, 1984; Fertil and Malaise, 1985; Tucker and Thames, 1989; Brock et al, 1990, 1992; Davidson et al, 1990; West and Hendry 1992; West et al, 1993, 1997; Girinsky et al, 1993, 1994).

In this study an examination has been made of the radiosensitivity of head and neck cancers using a clonogenic assay. We have shown that in vitro growth of tumours from the head and neck region can be achieved using a soft-agar assay. The culturing success rate was 74% and $SF_2$ was obtained for 63% of all patients biopsied. The latter figure is similar to the 60% success rate reported by Brock et al (1990) for primary head and neck carcinomas using the CAM assay. Girinsky et al (1993) reported a success rate for obtaining $SF_2$ values in head and neck cancers using the CAM assay of 75% when the cell yield was high enough to allow cell cultures to be set up. Using the same criteria we also obtained a success rate of 74% in obtaining $SF_2$ measurements.

Using a soft-agar assay, tumour cells grow as spherical colonies in an agar layer while the growth of cells that require anchorage to a solid substrate such as fibroblasts is inhibited. However, there have been reports recently that have shown that fibroblasts can grow in soft agar (Parkins and Steel, 1990; Lawton et al, 1994; Stausbol-Gron et al, 1995). In the present study, fibroblast growth was not inhibited as a small number of large, star-shaped and vimentin-positive colonies was identified. No difficulties were found in the discrimination of such colonies and tumour. Unfortunately fibroblast colony numbers were too small to allow determination of fibroblast $SF_2$ and the colonies were all excluded in this study.

Other assays for measuring radiosensitivity are being investigated to increase both the assay time and success rate (see introduction) but none are suitable yet for routine clinical application. To date the strongest correlation between tumour radiosensitivity measurements and clinical outcome has been achieved using a soft-agar assay on cervix tumours treated with radical radiotherapy (West et al, 1993, 1997). Girinisky et al (1993, 1994) also reported a significant but less strong relationship using the CAM assay in head and neck tumours treated predominantly with radiotherapy alone. It may be that by using a soft-agar assay a significant relationship might be found between tumour radiosensitivity and clinical outcome for cancers treated with post-operative radiotherapy. Therefore, despite the fact that Brock et al (1992) failed to show a relationship between tumour radiosensitivity measured using the CAM assay and clinical outcome in patients treated with radiotherapy plus surgery, there is interest in repeating his study using a soft-agar clonogenic assay. As soon as we have adequate follow-up, we will report on the correlation between $SF_2$ and outcome.

Using a soft-agar assay our mean value for the radiosensitivity of head and neck cancers was higher than those reported using the CAM assay (Brock et al, 1990; Girinsky et al, 1994) and this probably reflects differences between the two assays. The radiosensitivites of the head and neck cancers studied were variable ($SF_2$ from 0.10 to 1.00). The latter observation supports the idea that a predictive assay based on tumour $SF_2$ measurements might be useful in head and neck cancers. In addition, we have found that the radiosensitivity of head and neck cancers is independent of many clinical parameters, i.e. stage, nodal status and patient age, and this confirms the findings of others (Brock et al, 1992; Girinsky et al, 1993, 1994). This study also confirmed the finding that there are no significant differences in mean radiosensitivity for tumours of different sites within the head and neck region (Girinsky et al, 1993; Pekkola-Heino et al, 1995). The latter observation, however, is in contrast to a report by Brock et al (1992), who found that oral cavity tumours were more radioresistant than carcinomas of the larynx, an observation that is consistent with clinical observations (Corvò et al, 1994). Primary tumour growth pattern scored as exophytic or ulcerating is clinically believed to represent different degrees of tumour oxygenation. We found no relationship between tumour radiosensitivity and growth pattern.

© Cancer Research Campaign 1998

We also found no relationship between tumour radiosensitivity and CFE, which confirms previous findings in a number of different tumour types (West et al, 1991; Girinsky et al, 1994).

This study has also shown that head and neck and cervix tumours have similar radiosensitivity as reported by Girinsky et al (1993) using the CAM assay. Previous studies have suggested that there may be differences in the radiosensitivity of *primary* tumours (Rofstad et al, 1987; Brock et al, 1989). However, for the first time, we report that there are *significant* differences in the radiosensitivity of different tumour types that can be measured in primary cultures without establishing cell lines.

The ultimate aim of this study is to correlate the radiosensitivity data with clinical outcome for a tumour type in which radiosensitivity could have a clear role to play in determining the optimum primary treatment (surgery/radiotherapy). This will require adequate follow-up, a minimum of 2 years, for all the patients studied and will be carried out in the future. However, as there is a lack of clinical measurements of radiosensitivity in primary human tumours, our preliminary data are presented here. This work has shown the feasibility of measuring head and neck cancer radiosensitivity using a soft-agar clonogenic assay and that using this assay the radiosensitivity is independent of many clinical features and is similar to that of cervix carcinomas. In addition, we have shown, in *primary* tumours, that cancers differ significantly in intrinsic radiosensitivity.

### ACKNOWLEDGEMENTS

This work was supported by the King Gustav V Jubilee Clinic Cancer Research Foundation in Gothenburg and Berit and Carl Johan Wettergrens Foundation for Cancer Research in Sweden. Catharine West is supported by the Cancer Research Campaign (UK). The authors thank Ms Ragnhild Bernefors and Ms Ingegerd Hermansson for their dedicated helpfulness and practical assistance and PhD K-A Johansson at the Department of Radiophysics at Sahlgrenska University Hospital for his great assistance with the dosimetry.

### REFERENCES

Bristow RG and Hill RP (1990) Comparison between *in vitro* radiosensitivity and *in vivo* radioresponse in murine tumour cell lines. II. *In vivo* radioresponse following fractionated treatment and *in vitro/in vivo* correlations. *Int J Radiat Oncol Biol Phys* **18**: 133–145

Brock WA, Baker FL and Peters LJ (1989) Radiosensitivity of human head and neck squamous cell carcinomas in primary culture and its potential as a predictive assay of tumour radiocurability. *Int J Radiat Biol* **56**: 751–760

Brock WA, Baker FL, Wike JL, Sivon SL and Peters LJ (1990) Cellular radiosensitivity of primary head and neck squamous cell carcinomas and local tumor control. *Int J Radiat Oncol Biol Phys* **18**: 1283–1286

Brock WA, Brown BW, Goefpert H and Peters LJ (1992) *In vitro* radiosensitivity of tumor cells and local tumor control by radiotherapy. In *Radiation Research: A Twentieth Century Perspective*, Dewey WC, Edington M, Fry RJM, Hall EJ and Whitmore GF (eds), pp. 696–699. Academic Press: San Diego

Coco-Martin JM, Smeets MF, Poggensee M, Mooren E, Hofland I, van den Brug M, Ottenheim C, Bartelink H and Begg AC (1994) Use of fluorescence *in situ* hybridization to measure chromosome abberations as a predictor of radiosensitivity in human tumour cells. *Int J Radiat Biol* **66**: 297–307

Corvò R, Sanguineti G, Scala M, Garaventa G, Santelli A, Barbieri M and Vitale V (1994) Primary site as predictive factor of local control in advanced head and neck tumors treated by concomitant boost accelerated radiotherapy. *Tumori* **80**: 135–138

Davidson SE, West CML, Roberts SA, Hendry JH and Hunter RD (1990) Radiosensitivity testing of primary cervical carcinoma – evaluation of intra- and inter-tumour heterogeneity. *Radiother Oncol* **18**: 349–356

Deacon J, Peckham MJ and Steel GG (1984) The radioresponsiveness of human tumours and the initial slope of the cell survival curve. *Radiother Oncol* **2**: 317–323

Fertil B and Malaise E-P (1985) Intrinsic radiosensitivity of human cell lines is correlated with radioresponsiveness of human tumours: analysis of 101 published survival curves. *Int J Radiat Oncol Biol Phys* **11**: 1699–1707

Girinsky T, Lubin R, Pignon JP, Chavaudra N, Gazeau J, Dubray B, Cosset JM, Socie G and Fertil B (1993) Predictive value of *in vitro* radiosensitivity parameters in head and neck cancers and cervical carcinomas: preliminary correlations with local control and overall survival. *Int J Radiat Oncol Biol Phys* **25**: 3–7

Girinsky T, Bernheim A, Lubin R, Tavakoli-Razavi T, Baker F, Janot F, Wibault P, Cosset J-M, Duvillard P, Duverger A and Fertil B (1994) In vitro parameters and treatment outcome in head and neck cancers treated with surgery and/or radiation: cell characterization and correlations with local control and overall survival. *Int J Radiat Oncol Biol Phys* **30**: 789–794

Lawton PA, Hodgkiss RJ, Eyden BP and Joiner MC (1994) Growth of fibroblasts as a potential confounding factor in soft-agar clonogenic assays for tumour cell radiosensitivity. *Radiother Oncol* **32**: 218–225

Parkins CS and Steel GG (1990) Growth and radiosensitivity testing of human tumour cells using the adhesive tumour cell culture system. *Br J Cancer* **62**: 935–941

Pekkola-Heino K, Jaakkola M, Kulmala J and Grenman R (1995) Comparison of cellular radiosensitivity between different localizations of head and neck squamous-cell carcinoma. *J Cancer Res Clin Oncol* **121**: 452–456

Ramsay J, Ward R and Bleehen NM (1992) Radiosensitivity testing of human gliomas. *Int J Radiat Oncol Biol Phys* **24**: 675–680

Rofstad EK, Wahl A and Brustad T (1987) Radiation sensitivity *in vitro* of cells isolated from human tumor surgical specimens. *Cancer Res* **47**; 106–110

Schwartz JL, Mustafi R, Beckett MA and Weichselbaum (1996) DNA double-strand break rejoining rates, inherent radiation sensitivity and human tumour response to radiotherapy. *Br J Cancer* **74**: 37–42

Spiessl B, Beahrs OH, Hermanet P, Hutter RVP, Scheibe O, Sobin LH and Wagner G (1989) *International Union against Cancer INM Atlas Illustrated Guide to the TNM/pTNM Classification of Malignant Tumours*, pp. 4–49 Springer Verlag: Berlin

Stausbol-Gron B, Nielsen OS, Moller Bentzen S and Overgaard J (1995) Selective assessment of *in vitro* radiosensitivity of tumour cells and fibroblasts from single tumour biopsies using immunocytochemical identification of colonies in the soft-agar clonogenic assay. *Radiother Oncol* **37**: 87–99

Tucker SL and Thames HD (1989) The effect of patient-to-patient variability on the accuracy of predictive assays of tumour response to radiotherapy – a theoretical evaluation. *Int J Radiat Oncol Biol Phys* **17**: 145–157

West CML (1995) Invited review: intrinsic radiosensitivity as a predictor of patient response to radiotherapy. *Br J Radiol* **68**: 827–837

West CML and Hendry JH (1992) Intrinsic radiosensitivity as a predictor of patient response to radiotherapy. *Br J Radiol* **Supplement 24**: 146–152

West CML, Davidson SE, Pool C, James RD and Schofield PF (1991) Lack of a relationship between colony-forming-efficiency and surviving fraction at 2 Gy. *Radiat Res* **126**: 260–263

West CML, Davidson SE, Roberts SA and Hunter RD (1993) Intrinsic radiosensitivity and prediction of patient response to radiotherapy for carcinoma of the cervix. *Br J Cancer* **68**: 819–823

West CML, Davidson S, Roberts SA and Hunter RD (1997) The independence of intrinsic radiosensitivity as a prognostic factor for patient response to radiotherapy of carcinoma of the cervix. *Br J Cancer* (in press)

Zaffaroni N, Orlandi L, Villa R, Bearzatto A, Rofstad EK and Silvestrini R (1994) DNA double-strand break repair and radiation response in human tumour primary cultures. *Int J Radiat Biol* **66**: 279–285

Zolzer F, Alberti W, Pelzer T, Lamberti G, Hulskamp FH and Streffer C (1995) Changes in S-phase fraction and micronucleus frequency as prognostic factors in radiotherapy of cervical carcinoma. *Radiat Oncol* **36**: 128–132

© Cancer Research Campaign 1998